# National Public Health Dashboards: Protocol for a Scoping Review

**DOI:** 10.2196/52843

**Published:** 2024-05-16

**Authors:** Itzhak Yanovitzky, Gretchen Stahlman, Justine Quow, Matthew Ackerman, Yehuda Perry, Miriam Kim

**Affiliations:** 1 School of Communication & Information Rutgers University New Brunswick, NJ United States; 2 School of Information Florida State University Tallahassee, FL United States

**Keywords:** dashboard, scoping review, public health, design, development, implementation, evaluation, user need, protocol, data dashboards, audiences, audience, systematic treatment, public health data dashboards, PRISMA-ScR, snowballing techniques, gray literature sources, evidence-informed framework, framework, COVID-19, pandemic

## Abstract

**Background:**

The COVID-19 pandemic highlighted the importance of robust public health data systems and the potential utility of data dashboards for ensuring access to critical public health data for diverse groups of stakeholders and decision makers. As dashboards are becoming ubiquitous, it is imperative to consider how they may be best integrated with public health data systems and the decision-making routines of diverse audiences. However, additional progress on the continued development, improvement, and sustainability of these tools requires the integration and synthesis of a largely fragmented scholarship regarding the purpose, design principles and features, successful implementation, and decision-making supports provided by effective public health data dashboards across diverse users and applications.

**Objective:**

This scoping review aims to provide a descriptive and thematic overview of national public health data dashboards including their purpose, intended audiences, health topics, design elements, impact, and underlying mechanisms of use and usefulness of these tools in decision-making processes. It seeks to identify gaps in the current literature on the topic and provide the first-of-its-kind systematic treatment of actionability as a critical design element of public health data dashboards.

**Methods:**

The scoping review follows the PRISMA-ScR (Preferred Reporting Items for Systematic Reviews and Meta-Analyses Extension for Scoping Reviews) guidelines. The review considers English-language, peer-reviewed journal papers, conference proceedings, book chapters, and reports that describe the design, implementation, and evaluation of a public health dashboard published between 2000 and 2023. The search strategy covers scholarly databases (CINAHL, PubMed, Medline, and Web of Science) and gray literature sources and uses snowballing techniques. An iterative process of testing for and improving intercoder reliability was implemented to ensure that coders are properly trained to screen documents according to the inclusion criteria prior to beginning the full review of relevant papers.

**Results:**

The search process initially identified 2544 documents, including papers located via databases, gray literature searching, and snowballing. Following the removal of duplicate documents (n=1416), nonrelevant items (n=839), and items classified as literature reviews and background information (n=73), 216 documents met the inclusion criteria: US case studies (n=90) and non-US case studies (n=126). Data extraction will focus on key variables, including public health data characteristics; dashboard design elements and functionalities; intended users, usability, logistics, and operation; and indicators of usefulness and impact reported.

**Conclusions:**

The scoping review will analyze the goals, design, use, usefulness, and impact of public health data dashboards. The review will also inform the continued development and improvement of these tools by analyzing and synthesizing current practices and lessons emerging from the literature on the topic and proposing a theory-grounded and evidence-informed framework for designing, implementing, and evaluating public health data dashboards.

**International Registered Report Identifier (IRRID):**

DERR1-10.2196/52843

## Introduction

### Background

The disjointed public health response to the COVID-19 pandemic highlighted the critical importance of having robust public health data systems in place and the potential utility of data dashboards for ensuring timely and unrestricted access to critical public health data. Data dashboards have been used extensively in the pandemic, collating real-time public health data, including confirmed cases, deaths, and testing figures, to keep the public informed and support policymakers in refining interventions [1,2]. The growing availability of data visualization platforms and tools, coupled with the ubiquitous use of dashboards to chronicle different aspects of the COVID-19 pandemic, increased the appeal of data dashboards to a wide and diverse range of decision makers including public health leaders and professionals, health care providers, community leaders, policymakers, and advocates [3,4]. Data dashboards are frequently touted as cost-effective means to share and access public health and other types of publicly available data because they integrate and transform complex data into intuitive information displays, afford immediate availability and near-universal access of multiple and diverse groups of users to data, and allow users to explore data on their own to answer questions that are important to them [5-8]. They are also increasingly recognized for their democratizing potential, both in terms of making data available to a wider and more diverse range of audiences and ensuring that diverse stakeholders, particularly those who are less privileged and are most likely to be impacted by how data are interpreted and used in decision-making, have the power and opportunity to shape what and how data are used in this context, thus reframing how we think about health disparities and social determinants of health [9].

As public health data dashboards are poised to become more ubiquitous, it is imperative to proactively consider how they may be best integrated with data systems and decision-making routines of diverse audiences to advance sound, equitable, and sustainable public health policies and practices [3,10]. Getting there likely requires additional investments in the continued development, improvement, and sustainability of these tools, but progress in this direction is currently impeded by considerable fragmentation in the academic literature regarding the purpose (why) and intended audiences (who) of public health data dashboards, the design philosophy and features (what) that enable informed and consistent use of these tools across user populations and decision-making contexts, the causal mechanisms (how) that link use of public health data dashboards to users’ decisions and actions, and the factors (conditions, circumstances, and support mechanisms) that explain variations in use and usefulness of these tools across users and applications [3,8,10,11]. A systematic review and synthesis of the extant literature on this topic that is focused on closing these gaps can therefore be extremely valuable for developing a theory-grounded and evidence-informed framework to guide the design, implementation, and evaluation of effective public health data dashboards.

### Aims and Prior Work

This scoping review will provide a descriptive and thematic overview of the purpose; intended audiences; health topics; design elements and characteristics; evidence of the impact of national public health data dashboards; and the processes used for development, implementation, and evaluation. Previous reviews of the literature on this topic have focused on identifying and evaluating key design features of public health data dashboards, but most were limited to a specific health topic, such as COVID-19 [2,12], food and nutrition systems [13], infectious diseases [14], and environmental hazards [15], or were limited in focus to specific design features such as data visualization design [16] or usability and usefulness [4]. By comparison, the planned scoping review will be much broader and comprehensive in terms of the scope of health topics and applications considered, but also in terms of considering different potential goals of data dashboards (eg, alert, educate, and persuade), theories of action (or how dashboards are presumed or expected to work), and outcomes of use (including impact indicators)—and comparing these across different settings and intended audiences.

In addition, this scoping review is poised to provide the first-of-its-kind systematic treatment of actionability as a critical design element of public health data dashboards. Stimulated by disjointed public health response to the COVID-19 pandemic, there has been a growing interest in the question of what makes public health data dashboards actionable, that is, ensuring they provide an optimal match for both purpose and use of data in support of decisions that lead to sound and equitable public health policies and practices [17]. Yet, actionability, as applied to public health data dashboards, is not yet fully defined or sufficiently operationalized to inform the design and implementation of such tools. Ivanković et al [18], for example, defined data dashboard actionability according to 7 features: (1) knowing and clearly stating the desired consumers of the information, (2) selecting and presenting appropriate indicators, (3) clearly stating the sources of data and methods used to generate indicators, (4) demonstrating variation over time and linking changes to public health interventions, (5) providing as high a spatial resolution as possible to enable consumers to evaluate local risk, (6) disaggregating data to population subgroups to further enable evaluation of risk, and (7) providing narrative information to enhance interpretation of the data by the consumer. This type of user-centered conception understands actionability as a function of both usability and degree of match between data and users’ information needs is intuitive but may not be adequate or sufficient to assess the actionability of dashboards intended for a general audience [19]. Other scholars in this space considered a design-centered conception of actionability [20]. In their view, to be actionable, dashboards must prompt or trigger users to act on data by being integrated, via behavioral design, into users’ data use practices or routines such as assessing performance on tasks or progress on goals. Finally, some advocate for a decision-centered conception of actionability, whereby data dashboards are considered actionable to the extent they provide data, analyses, and forecasts (eg, predictive analytics) that allow decision makers to make an informed choice among alternatives [19,21,22]. All 3 conceptions appear to be relevant to the definition and operationalization of actionability as a key design feature of public health data dashboard and the scoping review will be instrumental both in terms of more fully explicating actionability based on the integration of existing conceptions, as well as identifying additional potential dimensions that may be used to this end.

Accordingly, the key research questions that will be addressed by this study are as follows: (1) What is the current landscape of national public health data dashboards? Who creates them, for what purpose, with what data, and for whom? (2) What processes and frameworks are used for the development, implementation, and evaluation of national public health data dashboards? What are common metrics or indicators for assessing use and impact? (3) What design approaches, principles, and features are most frequently incorporated in national public health data dashboards? How may they be associated with the actionability of these tools?

## Methods

### Study Design

Given the aims of this study and the considerable diversity in research questions and methodologies used across disciplines and fields to study public health data dashboards, a scoping review of the literature is a sound choice. A scoping review is a type of evidence synthesis that aims to identify and map relevant scholarship that meets predetermined inclusion criteria regarding the topic, field, context, concept, or issue under review [23]. Like other types of systematic reviews, rigorous scoping reviews are based on well-defined methodological guidance and reporting standards that include a priori protocol, eligibility criteria, and a comprehensive search strategy. Accordingly, this study will follow the PRISMA-ScR (Preferred Reporting Items for Systematic Reviews and Meta-Analyses Extension for Scoping Reviews), which is the most up-to-date and advanced approach for conducting and reporting scoping reviews [24].

### Selection Criteria and Search Strategy

For the purposes of this scoping review, we define the “public health data dashboard” as a publicly accessible, web-based, and regularly updated information management and data visualization tool that displays and tracks certain public health indicators, metrics, and data points that can support decisions regarding population health. This definition is inclusive of a broad range of population health-relevant data such as vital statistics, epidemiological surveillance, aggregated measures of access and use of health services, community health indicators, and health information ecology (eg, data that tracks the spread of misinformation about a health topic), but excludes the use of data dashboards in clinical and health care organizations (eg, data used to track or benchmark internal performance or practices), as well as dashboards incorporated into patient portals.

Accordingly, the target population of this scoping review consists of all English-language, full-text, peer-reviewed journal papers, conference proceedings, book chapters, and reports that describe the design, implementation, and evaluation of a public health dashboard published between 2000 and 2023. Whereas the rapid advancements in dashboard technology in recent years may warrant a greater focus on more recent research, adopting a broader historical perspective can be useful for determining what, if anything, changed over time regarding the design philosophies and theories of action guiding the development and implementation of these tools. For the same reason, no geographical location, health focus, or methodological orientation–based restrictions will be imposed as selection criteria. Research reports involving data dashboards that do not use national data sources (eg, regional, state, or county level) are beyond the scope of the current review as prior research suggests that published case studies of these types of dashboards are not comparable given considerable variations in the resources available to develop and maintain data dashboards, availability and quality of data, and intended audiences [25]. However, we recognize that such case studies can contribute valuable insights regarding the actionability of public health data dashboards despite being underrepresented in the current literature on the topic [3]. Accordingly, we plan to compare and contextualize the findings of this scoping review against the findings and conclusions of several recent studies that systematically assessed key design and use elements of state-level data dashboards [25-27]. In addition, the next phase of this project, which involves the mapping and analysis of publicly accessible national and state-level US public health data dashboards, was intentionally designed to produce a more systematic and complete account of similarities and differences among public health data dashboards across levels.

Our search procedure is designed to minimize potential errors in our search strategies that negatively affect the quality and validity of this scoping review [28]. First, in collaboration with a research librarian, we searched both the Medical Subject Headings database and keywords listed in recently (2019 and onward) published journal papers on the topic of public health data dashboards to identify the most relevant keywords and terms for searching for relevant publications that meet our inclusion criteria. In the next step, we followed an established procedure [29] to experiment with different combinations of databases and search queries to optimize the recall (sensitivity) and precision (specificity) of our search strategy. Given the aims of this scoping review, we opted for a search strategy that maximizes coverage, that is, will increase the likelihood of identifying all or as many as possible relevant resources. Hence, the reviewer needs to select a search system that provides the best coverage of the chosen search topic. Accordingly, we searched CINAHL, PubMed, Medline, and Web of Science databases in June 2023 for published research reports using the search query ([“dashboard” OR “data dashboard” OR “Information visualization” OR “data visualization”] AND [“public health” OR “population health”]). These databases were selected because they were identified, via rigorous testing, as providing optimal coverage of research published across a broad range of disciplines and fields [30]. In our testing, this combination of databases and search queries increased recall but resulted in a precision level of about 25%, providing a rough estimate of the expected number of relevant documents returned by the search. We conducted supplementary searches of gray literature using the same search query to search OpenGrey for additional documents that met all selection criteria.

### Study Selection

All papers retrieved by the search across the 4 databases were imported into and initially reviewed using Zotero (Corporation for Digital Scholarship), a free and open-source reference management software to manage bibliographic data. After duplicate records were identified and duplicates removed, the remaining pool of documents was manually screened by members of the research team for relevance. All coders (n=5) first received training on the task and then were provided with a random sample of 45 records to screen for relevance by applying the selection criteria. Agreement among coders was assessed using Krippendorff α [31], and the test result was significantly lower (α=0.37) than the acceptable standard (α=0.70). Coders then received additional training on the task of screening items for relevance and then independently coded a fresh set of 25 randomly selected items. An intercoder agreement was reassessed and reached an acceptable standard (α=0.78), allowing coders to proceed with the task, with any potential ambiguity regarding relevance resolved via a full team review.

### Data Charting

A preliminary list of data elements for charting is presented in Textbox 1, but an iterative process will be used to identify additional elements for data extraction and analysis as the study progresses and based on inputs received from the project’s expert advisory group (composed of national data dashboard creators). A standardized data extraction form will be developed and pilot-tested by following the same procedure described above for validating the screening and selection procedure including tests of intercoder agreement. Once a high level of agreement is achieved, coders will proceed to extract the data from all documents included in this scoping review. Any confusion or disagreement regarding data extraction will be resolved by discussion among research team members.

**List of data extraction elements.**

**Study identifiers**

- Metadata (title, authors, journal, year of publication, and keywords)
- Study type (eg, descriptive, exploratory, and explanatory)
- Research methodology
- Study focus (eg, development, implementation, and evaluation)
- Geographic location (country)

**Data characteristics**

- Data sources
- Health topics
- Type of data (eg, epidemiological, health services, and behavioral)
- Populations represented in the data
- Indicators or metrics selected for visualizations
- Data level of granularity (eg, national, state, county, and city)

**Dashboard design characteristics**

- Stated goals or purposes of the dashboard (eg, tracking or monitoring)
- Design philosophy cited (eg, user-friendly, functional, and co-design)
- Design process (eg, iterative and collaborative)
- Dashboard features (eg, customization and search functionalities)
- Data visualization tools (eg, maps, graphs, and tables)

**Users and usability**

- Intended audiences
- Public access (open, restricted/limited, requires registration)
- Dissemination channels (eg, social media, news outlets, email, and listserv)
- Reported use- or usability-related barriers or challenges

**Logistics or operation**

- Ownership or hosting
- Source of funding
- Software tools (commercial and open source)
- Data updating and quality assurance protocols
- Technical support (eg, user manuals, training, and customer service option)

**Performance and usefulness or impact evaluation**

- Evaluation methodology
- Use or usability indicators captured (eg, website analytics and user ratings)
- Impact indicators or other evidence of impact
- Explanations given for observed effects or impact (or lack of)

### Data Collection, Curation, and Analysis

Data extraction and collection will be performed using a web-based survey tool (Qualtrics; Silver Lake Technology Management, LLC) as, in our experience, this method improves workflow and reduces recording errors compared with alternative methods (eg, entering data in a spreadsheet). The data file generated will be cleaned and then converted to an SPSS (IBM Corp) file for data analyses (mainly frequency counts and cross-tabulation of variables). This scoping review will follow the PRISMA-ScR checklist [24] for reporting methods and outcomes.

## Results

**Figure 1:**
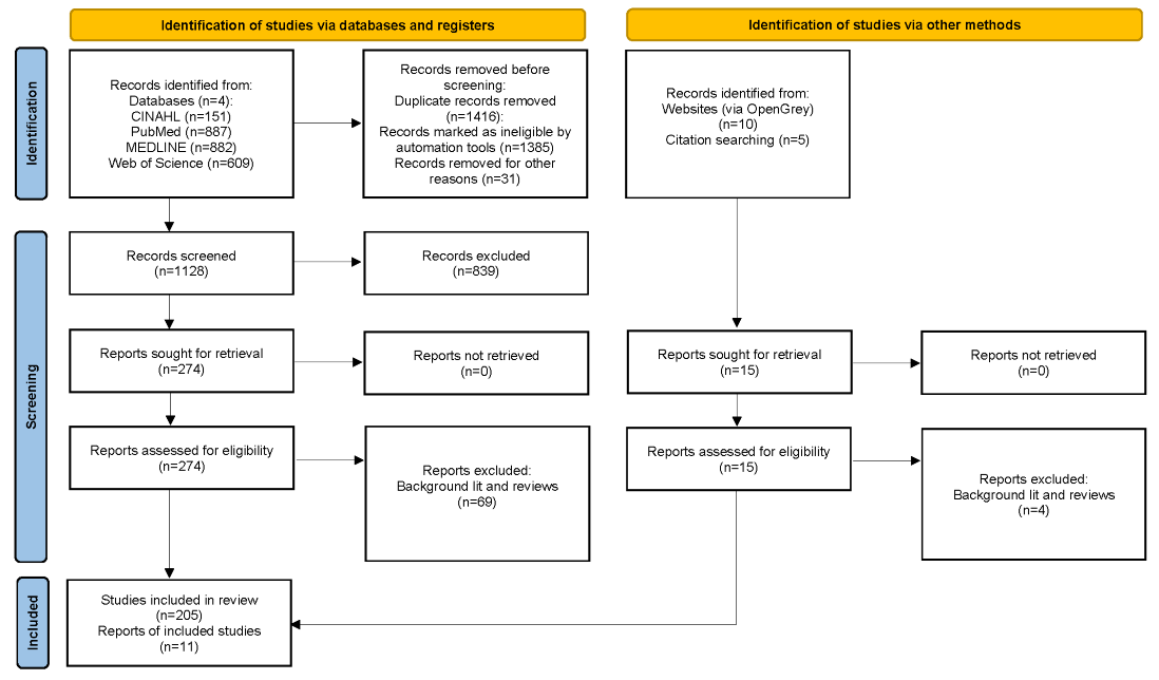
Flow diagram of paper screening and selection process [33].

In June 2023, electronic database searches for relevant papers were completed using the search procedure outlined above. After the removal of duplicate results, the remaining records were screened manually by members of the research team following the procedure outlined above. The PRISMA flow diagram (Figure 1) summarizes the process and outcomes of the screening process. As shown, a total of 2529 documents (peer-reviewed journal papers, conference proceedings, and book chapters) were initially retrieved. An automated Zotero plugin was initially used to remove duplicate records (n=1385), leaving 1144 records. A manual quality control screening identified additional duplicate records (primarily preprint and published versions of the same work), leaving 1113 records. Following the addition of “grey literature” sources (n=10) and additional papers that were identified through our snowballing review of sources cited in other related literature reviews (n=5) [2,4,12,32], the corpus included 1128 documents. Of these, a total of 289 documents (or 25.6% of all documents screened) met the study’s selection criteria and were retained for analysis. This percentage is consistent with the estimate of precision we produced (25%) based on our initial experimentation and testing of our search strategy. These documents can be divided into three general categories of research studies: (1) US case studies of national public health data dashboard (n=90), (2) non-US case studies of national public health data dashboards (n=127), and (3) reviews of the literature and other background information items such as expert evaluations of dashboard design elements that are not specific to a particular data dashboard (n=73), which are therefore excluded (but will be consulted for comparing and contextualizing the scoping review findings), leaving a total of 216 case studies included in the review. We will conduct an initial round of review to determine whether and how differences across case studies may influence the validity and reliability of the findings and the conclusion drawn from this scoping review before deciding on the final pool of papers to be coded and analyzed. We aim to finish the coding and analysis of papers and draft the final report by mid-2024. Findings will be summarized narratively (with the addition of summary tables and graphs) and organized around the research questions motivating the review. The final report will be submitted for publication along with the completed PRISMA-ScR reporting checklist.

## Discussion

### Principal Findings

As public health data dashboards are poised to become more ubiquitous, it is imperative to proactively consider how they may be best designed to leverage public health data systems and meet the information needs of diverse audiences to support sound decisions regarding equitable and sustainable public health policies and practices [3,10]. However, the scientific literature available to inform such efforts is considerably fragmented, lacking a standard, coherent focus regarding the goals, design, use, usefulness, and impact of these tools, as well as regarding factors (ie, conditions, circumstances, and support mechanisms) that explain variations in their use and usefulness across users and applications [3,8,10,11]. We intended to use the findings of this scoping review to inform the development of a theory-grounded and evidence-informed framework for guiding the design, implementation, and evaluation of effective public health data dashboards. In addition, the review will produce evidence that can be used for identifying important gaps and deficiencies, but possibly also best practices, in current practices that can enhance the efficient and effective use of these tools moving forward.

### Limitations

The scoping review methodology used in this study has several limitations. First, whereas we took multiple steps to ensure the rigor of our literature search and screening strategy, it is still reasonable to assume that some relevant studies are overlooked. However, by opting for a procedure designed to maximize coverage at the expense of precision, we are potentially able to mitigate this limitation. Second, because the studies included in the review vary considerably in the type and depth of the information provided, data extraction and analysis may not be sufficiently robust to support sound conclusions and recommendations based on findings. We will take care to qualify any conclusions or recommendations accordingly and to reflect critically on the state of research on this topic. Third, it is possible for potential bias in findings and conclusions to creep in because studies that considered a particular type of data dashboard are disproportionately represented in the literature on the topic, for example, studies of COVID-19 data dashboards [4]. If this is the case, we will make sure to minimize bias by clustering dashboards of the same type (including multiple studies of the same data dashboard) and analyzing them separately. Fourth, limiting the scope of the review to national public health data dashboards may result in potentially missing valuable insights that can be drawn from case studies of regional, state, and county-level data dashboards. However, such insights may be gleaned from recently published work that systematically collected and analyzed such case studies [25-27] and will also emerge from the next phase of this research project (mapping and analysis of the national and state-level public health data dashboards ecosystem in the United States).

### Conclusions

Public health data dashboards have significant potential to ensure timely and unrestricted access to critical public health data that can inform decisions made by a wide range of stakeholders and decision-makers. This scoping review will inform the continued development and improvement of these tools by analyzing and synthesizing current practices and lessons emerging from the literature on the topic and proposing a theory-grounded and evidence-informed framework for designing, implementing, and evaluating public health data dashboards.

## Abbreviations

PRISMA-ScR: Preferred Reporting Items for Systematic Reviews and Meta-Analyses Extension for Scoping Reviews

## Data Availability

The data sets generated or analyzed during this study are available from the corresponding author on reasonable request.
